# Trend in eating habits among Lithuanian school-aged children in context of social inequality: three cross-sectional surveys 2002, 2006 and 2010

**DOI:** 10.1186/1471-2458-12-52

**Published:** 2012-01-19

**Authors:** Apolinaras Zaborskis, Reda Lagunaite, Ryan Busha, Jolita Lubiene

**Affiliations:** 1 Lithuanian University of Health Sciences, Academy of Medicine, Faculty of Public Health, Lithuania; 2 Utena University of Applied Sciences, Faculty of Health Care and Social Care, Lithuania

## Abstract

**Background:**

Intermittent monitoring of food intake at the population level is essential for the planning and evaluation of national dietary intervention programs. Social-economic changes in Lithuania have likely affected dietary habits, but only a limited number of temporal studies on food intake trends among young population groups have been published. The aim of this study was to investigate changes in eating habits among Lithuanian school-aged children from 2002 to 2010, and to explore the association of these changes with the respondents' reported socio-economic status (SES).

**Methods:**

We used Lithuanian data from the cross-national Health Behaviour in School-aged Children (HBSC) study collected in 2002, 2006 and 2010. Analyses were conducted on comparable questionnaire-based data from children aged 11, 13 and 15 (total n = 17,189) from a random sample of schools. A food frequency questionnaire was used to investigate frequencies of food consumption. Logistic regression was used to examine the affects of changing social variables on reported diet trends.

**Results:**

In Lithuania, school-aged children have low intakes of fruits and vegetables. Only 21.1% of boys and 27.1% of girls reported daily fruit consumption. Similarly, 24.9% of boys and 29.6% of girls disclosed vegetable intake at least once daily. Comparing 2010 to 2002, the proportion of girls who consumed fruits daily increased from 24.2% to 31.0% (p < 0.001) but the proportion of boys who consumed vegetables daily decreased from 29.3% to 23.1% (p < 0.001). In 2006, for both sexes, there were observed increases in regular (at least five days a week) intake of sweets and chocolates, biscuits and pastries, and soft drinks; however, in the next survey (2010) these figures decreased. In addition, between 2006 and 2010, a substantial decrease in regular consumption of chips and fast food was also detected. Fruit and vegetable consumption as well as intake of sweets and chocolates, biscuits and pastries and soft drinks increased with family social-economic status and family material wealth. Trends in consumption of fruits, and other foods, and their association with changing social variables were demonstrated using the ORs estimated by three logistic models, using 2002 as the reference point. Changes in social variables from 2002 to 2010 affected the likelihood of daily consumption of fruits among boys by 22.5% (the corresponding OR decreased from 1.11 to 0.86) and among girls by 34.0% (the corresponding OR decreased from 1.41 to 1.12). Over the study period, changing social variables had little impact on the daily consumption of vegetables and other foods.

**Conclusions:**

Based on the food consumption trends observed in Lithuania, increases in consumption of fruits and vegetables should be promoted, along with a reduction in the intake of less healthy choices, such as soft drinks and high-fat, high-sugar snack foods, by diminishing social inequalities in food consumption.

## Background

Diet is a major contributor to the development of chronic non-communicable diseases and other health problems. Assessment of temporal trends in dietary intake is essential for early detection of nutrition problems within entire populations [1]. Data from national surveys are the primary source of information for the planning and evaluation of national nutrition policies and dietary interventions and, in general, for the development of disease prevention and health promotion programs [2-4].

The need to monitor eating habits among young people has intensified in recent years due to the growing epidemic of overweight status worldwide [5,6]. Increases in other nutrition-related risk factors for chronic disease in children such as hypertension, hypercholesterolemia and Type 2 diabetes have also been observed [7,8]. Reports have suggested that diets low in fruits, vegetables and milk products or high in less healthy choices (including soft drinks and high-fat, high-sugar foods; and consumption of too much fat and saturated fat, and too little folate and calcium) increase the risk for overweight as well as prevalence of other risk factors [9].

In addition, growing evidence suggests that young people from developed countries are increasing making unhealthy food choices [6]. Although there are few sources of national data available on children's eating behaviours in Lithuania, information from the cross-national study on Health Behaviour in School-aged Children (HBSC) suggests that similar concerns exist for Lithuanian children, including low fruit and vegetable consumption and high consumption of sweets, chocolates, soft drinks and other less healthy choices [9]. Presently, schoolchildren nutrition is being discussed widely on the national health and education agendas [10].

Numerous studies have focused on social and economic determinants of healthy eating in the general population including children and adolescents [11]. A direct relationship was shown between food consumption patterns and income, food pricing, education, employment, product marketing, mass media and other determinants. In contrast to adults, children's eating behaviour is under the influence of a greater number of determinants. Children's dietary patterns evolve within a family context including family meals, parents' nutritional knowledge, parenting style, etc. The role of the school environment and the effect of national dietary guidelines on healthy eating in children and adolescents are considerable as well. Despite the growing interest in social and economic differences in food consumption behaviours, the number of studies among children and adolescents is limited; they are performed in few countries and often include non-representative samples. Moreover, comparison between these studies is difficult due to differences in the sampling method and the measurement of eating habits as well as social and economic variables.

Lithuania is located on the eastern shore of the Baltic Sea. It was part of the former Soviet Union for fifty years (1940-1990) and followed the typical eastern European economic, education, welfare and health care models. Since regaining its independence in March 1990, the country has begun a complex transition phase as it moves from a former totalitarian state, with a centralized economy, to a democratic society, with a market economy. This transition period in Lithuania, similar to other countries in Central and Eastern Europe, can be distinguished by its social and economic reforms, which have also influenced Lithuanian's nutrition habits [12].

Food prices are the most important consideration in food choice. Price determinants often lead to the selection of "cheaper" but biologically less valuable and spoiled foodstuffs in cases of restricted income. Part of the health consequences of these changes is clear now - the number of diseases caused by infected food is increasing. Other consequences will emerge in the decades to come. These negative effects are not acceptable for the population or the state. In order to prevent public health and social problems related to poor nutrition, it is crucial to identify and predict changes in eating behaviour of the population groups with special respect to children and adolescents.

The assessment of trends over time requires identical repeated measurements and study designs. However, these types of studies are difficult to continue in the long-term. We have access to comparable data on fruits, vegetables, and other food intakes from the cross-national HBSC study in Lithuania, where data has been collected since 1994. This study measured food intake using food frequency questionnaire methods; however, over time the phrasing of questions and response categories have changed, the most recent affecting our analysis came in 2002.

In the present study, we aim at describing trends in the prevalence of daily fruit and vegetable consumption and regular intake of low-nutritive foods (sweets, chips, soft drinks and other) among Lithuanian schoolchildren aged 11, 13 and 15 by cross-sectional data collected via three nationally representative and comparable questionnaire surveys in 2002, 2006 and 2010. Furthermore, we aim to demonstrate changes in social inequalities of food consumption and the contribution of social variables to the trends of food intake among the young Lithuanians.

## Methods

### Subjects and study design

The data presented here were obtained from three cross-sectional surveys conducted in 2002, 2006 and 2010 (March - April) in Lithuania according to the methodology of World Health Organization collaborative HBSC study (more detailed information about the study is provided elsewhere [9,13-15]). Researchers strictly followed the standardized international research protocol to ensure consistency in survey instruments, data collection and processing procedures.

The study conformed to the principles outlined in the Declaration of Helsinki and was approved by Kaunas Regional Committee for Biomedical Research (No BE-2-72, 30 Dec 2005 and No P1-170/2005, 12 Jan 2010). National and local educational institutions also agreed to the study protocols and procedures.

The population selected for sampling was 11, 13 and 15 years old attending school with the desired mean age for three age groups being 11.5, 13.5 and 15.5 respectively. Participants were selected using a clustered hierarchical sampling design, where the initial sampling unit was a class of the fifth, seventh or ninth grades (the most appropriate grades for required students' age groups). Samples of students were drawn to be representative by age and gender. The recommended sample size for each survey was approximately 1,500 students per age group [15]. In total, over 300 classes from approximately 100 Lithuanian schools, representing the entire country, were drawn for each survey to ensure the requested number of surveyed students.

Questionnaires were administered in school classrooms by form tutors who complied with written instructions. The time frame for filling out the questionnaires was 1 - 1 1/2 school period (45 - 70 min). Participants could freely choose to participate, and anonymity and confidentially were ensured at all stages of the study. After completing the questionnaires, students sealed the provided envelopes themselves with their questionnaires inside. Form tutors reported the number of participants and process of questioning. Response rates were over 90% during all three surveys.

Upon completion of the fieldwork, the data were prepared using standard methods and submitted to the HBSC International Data Bank at the Bergen University, Norway. The data were checked, cleaned, included in the international HBSC database, and returned to the country for further statistical processing.

The analysis presented here is based on the total number of 16,615 records (5,645, 5,632 and 5,338, respectively from the surveys of 2002, 2006 and 2010) selected by quality criteria of the international HBSC database.

### Measures

The questionnaire consisted of about 200 items assessing different aspects of health behaviour, subjective health, social and the psychological environment. Questionnaire topics and items were discussed and approved by the international experts involved in HBSC [15]. The national questionnaire was adopted after translation of the questionnaire from the Standard English version into Lithuanian and retranslation back into English.

In the present study, the outcome measure was a diet assessment with a self-administered food frequency scale, which included seven foods typically consumed by children and adolescents. Young people were asked how many times a week they drink soft drinks (coke or other soft drinks that contain sugar), eat fruits, vegetables, sweets and chocolates, biscuits and pastries, chips, and fast food items, e.g. hamburgers, doughnuts (the questions about chips and fast food were not asked in 2002). The possible responses were: "never", "less than once a week", "about once a week", "two to four days a week", "five to six days a week", "once a day, every day", and "every day, more than once". For fruit and vegetable intake, responses were dichotomized: less than daily and daily, whereas for the remaining food item responses were dichotomized as follows: less than five times a week and five or more days a week (in further analyses, the category "five or more days a week" was named by a "regular" term).

Four indicators of participant's social position used in all three surveys and were defined as follows: i) place of residence: urban area (the five largest cities of the country and towns such as regional centres) versus rural area (villages and countryside); ii) family structure: living in intact family (with both biological parents) versus living in non-intact family (missing a biological father or mother); iii) family socioeconomic status (SES), which was measured by a sum score of Family Affluence Scale and then defined low, middle and high family SES [16]; iv) subjective rating of family wealth into three categories: low ('not at all well off' or 'not so well off'), average and high ('very well off' or 'quite well off') according to the response options proposed for the question 'How well off do you think your family is?'

### Statistical analysis

Data for boys and girls were analyzed separately. Crude rates of dichotomized food intakes by social position groups were produced for each study year separately as well as for the total sample of 11-15 year olds surveyed in 2002, 2006 and 2010. We applied χ^2 ^and Z tests, where appropriate, to assess the differences in the prevalence of foods consumption in differing groups. For multivariate analyses, we used age, year of the survey and indicators of social position. Binary logistic regression analysis was used to produce odds ratios (OR) with 95% confidence intervals (CI), which indicated the likelihood of daily/regular eating of typical foods for boys and girls, with certain characteristics relative to the reference group.

Estimation of trends was based on analysis of changes in the annual rates of food intakes. Differences between years of the survey were also shown in the OR values, which were estimated by three logistic models:

1. Year of the survey + age

2. Year of the survey + age + family SES and subjective rating of family wealth

3. Year of the survey + age + family SES and subjective rating of family wealth + place of residence and family structure.

All models were adjusted by age of respondents, and 2002 (or 2006 for chips and fast food) denoted the reference year. To determine the statistical significance of the association between change of social factor and changes of eating frequency of a certain foodstuff, the relative reduction in OR for survey year caused by adding the factor to the model was calculated. Interactions between the survey years and other factors were tested.

All statistical analyses were performed with SPSS for Windows version 15.0 (SPSS Inc., Chicago, IL, USA) statistical software package. P value of ≤ 0.05 was considered statistically significant.

## Results

### Population characteristics

Table 1 shows the study population characteristics for boys and girls by survey year. It consisted of 51.4% (n = 8542) boys and 48.6% (n = 8073) girls. The distribution of students in the three age groups seemed equal. Such demographic characteristics varied little from survey to survey.

**Table 1:** General characteristics of the study samples of school-aged children in Lithuania by year of survey

Characteristic	No. (%) of respondents			
	
	2002 (N = 5645)	2006 (N = 5632)	2010 (N = 5338)	Total (N = 16615)
*Gender:*				
boys	2887(51.1)	2904(51.6)	2751(51.5)	8542(51.4)
girls	2758(48.9)	2728(48.4)	2587(48.5)	8073(48.6)
	χ^2 ^test: NS			

*Age group:*				
11-year-old	1867(33.1)	1864(33.1)	1845(34.6)	5576(33.5)
13-year-old	1873(33.2)	1907(33.9)	1734(32.5)	5514(33.2)
15-year-old	1905(33.7)	1861(33.0)	1759(33.0)	5525(33.3)
	χ^2 ^test: NS			

*Family structure:*				
intact family	4386(77.7)	3943(72.6)	3666(69.3)	12349(73.3)
non-intact family	1259(22.3)	1491(27.4)	1622(30.7)	4587(26.7)
missing		(198)	(50)	(248)
	χ^2 ^test: p < 0.001			

*Place of residence:*				
urban areas	2402(42.6)	2583(46.0)	2698(50.7)	7683(46.3)
rural areas	3243(57.4)	3036(54.0)	2627(49.3)	8906(53.7)
missing		(13)	(13)	(26)
	χ^2 ^test: p < 0.001			

*Family SES:*				
low	2880(53.1)	2084(37.6)	1176(22.6)	6140(38.0)
middle	2104(38.8)	2558(46.1)	2372(45.6)	7034(43.5)
high	443(8.2)	905(16.3)	1654(31.8)	3002(18.5)
missing	(218)	(85)	(136)	(439)
	χ^2 ^test: p < 0.001			

*Subjective rating of family wealth: *				
low	1654(29.4)	1136(20.4)	1050(19.8)	3840(23.3)
average	2799(49.8)	2703(48.6)	2638(49.7)	8140(49.4)
high	1169(20.8)	1723(31.0)	1615(30.5)	4507(27.3)
missing	(23)	(70)	(35)	(128)
	χ^2 ^test: p < 0.001			

More than a quarter (26.7%) of the children lived in non-intact families; this proportion grew significantly by study year. The numbers of surveyed children in urban and rural areas were almost equal.

According to our criteria for assessing family SES, 38.0%, 43.5% and 18.5% of families were identified as low, middle and high SES respectively. The change of these proportions over the study period demonstrates an increase in the numbers of families at higher SES positions. Part of this increase can be attributed to the growing number of home computers, as one of the measures on family SES was if the family owned one, two or more computers.

Analysis of the subjective rating of family wealth shows that about a half (49.4%) of students thought their family's wealth was about average, whereas approximately one-quarter considered their family poor (23.3%) or rich (27.3%). During the study period, positive changes in subjective rating of family's wealth were identified too, but generally between 2002 and 2006. Subjective rating of family wealth correlated positively with family SES; however, this correlation was not strong (r = 0.37; p < 0.01).

### Gender and age inequalities

**Figure 1:**
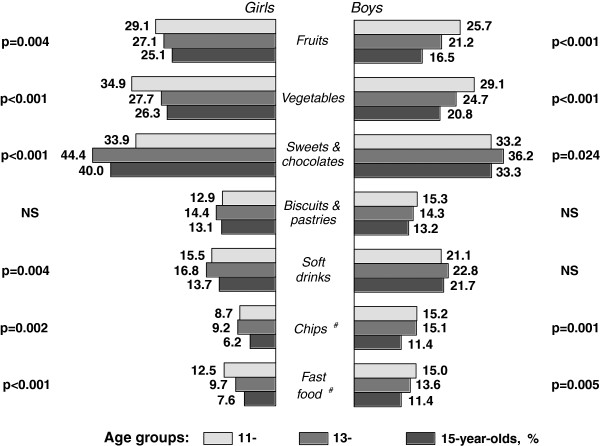
**Crude rates (%) of daily eating of fruits and vegetables, and regular intake of sweets and chocolates, biscuits and pastries, soft drinks, chips and fast food among boys and girls by age groups (pooled data from surveys in 2002, 2006 and 2010)**. p - significance from χ^2 ^test (NS - not significant); ^# ^no data from survey in 2002.

Girls at all ages were more likely to eat fruits and vegetables daily, as well as to consume sweets and chocolates on most days of the week (Figure 1). Overall, of the 11-15 year olds surveyed between 2002-2010, 21.1% of boys and 27.1% of girls (p < 0.001) reported eating fruit daily; 24.9% of boys and 29.6% of girls (p < 0.001) reported eating vegetables daily; and 34.3% of boys and 39.4% of girls (p < 0.001) reported eating sweets and chocolates at least five days a week. Daily fruit and vegetable consumption dropped significantly between the ages of 11 and 15 among both genders, while regular sweet and chocolate consumption was more common among 13 year olds.

Consumption of biscuits and pastries, soft drinks, chips and fast food increased among boys between 2002 and 2010. Overall (as mentioned above), 14.3% of boys and 13.5% of girls (NS) reported regular eating of biscuits and pastries, 21.9% of boys and 15.3% of girls (p < 0.001) reported regular drinking of soft drink, 13.9% of boys and 8.0% of girls (p < 0.001) reported regular chips consumption, and 13.4% of boys and 10.0% of girls (p < 0.001) reported regular eating of fast food. Regular intake of chips and fast food was less prevalent among 15-year-old students than among younger groups of respondents, whereas no difference was seen in eating of biscuits and pastries, and drinking of soft drinks (see Figure 1).

### Social inequalities

Table 2 presents association between eating habits and social factors by study population expressed as ORs (adjusted for age).

**Table 2:** Association between eating habits and social factors among school-aged children in Lithuania, pooled data from surveys in 2002, 2006 and 2010: odds ratio (OR) with 95% confidence interval (CI), adjusted for age

	Daily fruit intake	Daily vegetable intake	Regular intake of sweets and chocolates	Regular intake of biscuits and pastries	Regular drinking of soft drinks	Regular intake of chips^#^	Regular intake of fast food^#^
	OR 95% CI	OR 95% CI	OR 95% CI	OR 95% CI	OR 95% CI	OR 95% CI	OR 95% CI
**Boys:**							
*Place of residence:*							
urban area	1	1	1	1	1	1	1
rural area	**0.70** 0.63-0.77	**1.24** 1.12-1.37	**1.50** 1.37-1.64	**0.79** 0.70-0.89	**0.70** 0.63-0.78	0.94 0.81-1.09	1.03 0.88-1.20

*Family structure:*							
intact family	1	1	1	1	1	1	1
non-intact family	0.90 0.81-1.01	0.95 0.85-1.07	0.96 0.87-1.07	0.90 0.78-1.03	0.99 0.88-1.11	**1.19** 1.01-1.40	1.14 0.96-1.34

*Family SES:*							
low	1	1	1	1	1	1	1
middle	**1.55** 1.42-1.78	1.07 0.95-1.19	**1.25** 1.13-1.39	**1.20** 1.04-1.38	**1.16** 1.03-1.31	0.91 0.76-1.09	0.86 0.72-1.03
high	**2.15** 1.86-2.49	1.12 0.97-1.28	**1.48** 1.31-1.68	**1.38** 1.17-1.63	**1.39** 1.21-1.60	0.99 0.81-1.22	1.07 0.87-1.31

*Subjective rating of family wealth:*							
low	1	1	1	1	1	1	1
average	**1.28** 1.10-1.49	0.98 0.86-1.12	**1.38** 1.22-1.56	**1.27** 1.07-1.51	**1.24** 1.07-1.43	1.02 0.82-1.27	**0.74** 0.60-0.92
high	**1.97** 1.69-2.30	**1.20** 1.05-1.38	**1.64** 1.44-1.87	**1.60** 1.33-1.92	**1.92** 1.65-2.23	**1.37** 1.10-1.71	1.15 0.92-1.42

**Girls:**							
*Place of residence:*							
urban area	1	1	1	1	1	1	1
rural area	**0.66** 0.60-0.73	1.01 0.92-1.11	**1.77** 1.61-1.93	**0.70** 0.61-0.79	**0.74** 0.66-0.84	0.86 0.71-1.04	**1.24** 1.04-1.48

*Family structure:*							
intact family	1	1	1	1	1	1	1
non-intact family	0.90 0.81-1.01	**0.83** 0.75-0.93	0.94 0.85-1.04	1.05 0.91-1.21	1.12 0.98-1.28	**1.25** 1.02-1.54	1.21 1.00-1.46

*Family SES:*							
low	1	1	1	1	1	1	1
middle	**1.60** 1.42-1.78	1.07 0.96-1.19	**1.28** 1.16-1.41	1.13 0.98-1.30	1.09 0.95-1.24	**0.77** 0.62-0.96	**0.68** 0.57-0.82
high	**2.55** 2.22-2.93	**1.27** 1.11-1.45	**1.54** 1.35-1.75	1.20 1.00-1.45	1.11 0.94-1.33	0.79 0.60-1.03	**0.59** 0.45-0.76

*Subjective rating of family wealth:*							
low	1	1	1	1	1	1	1
average	**1.54** 1.36-1.75	1.01 0.90-1.14	**1.47** 1.31-1.64	**1.32** 1.12-1.55	**1.22** 1.04-1.42	0.93 0.72-1.19	**0.73** 0.58-0.90
high	**2.34** 2.03-2.69	**1.39** 1.22-1.59	**1.69** 1.49-1.92	**1.45** 1.21-1.74	**1.68** 1.42-1.99	1.03 0.79-1.36	0.87 0.68-1.10

^# ^no data from survey in 2002.

Urbanization was a significant determinant for students' eating behaviour. In all surveys boys and girls from rural areas indicated significantly less daily consumption of fruits (OR = 0.70 and OR = 0.66 respectively by gender), but boys from rural areas appeared more likely to eat vegetables daily (OR = 1.24). Regular consumption of sweets and chocolates was about 1.5 times more likely among adolescents from rural areas. Conversely, their peers from urban area were more likely to eat biscuits and pastries, and drink soft drinks. There were less significant associations for consumptions of chips and fast food by the living area variable.

Compared with the above variable, family structure might be seen as a less significant predictor of child eating behaviour, but some associations were notable. For instance, girls from non-intact families reported less daily consumption of vegetables (respectively 26.7% and 30.8%; OR = 0.83; p = 0.001), and also reported more frequent consumption of chips (respectively 9.1% and 7.5%; OR = 1.25; p = 0.035).

**Figure 2:**
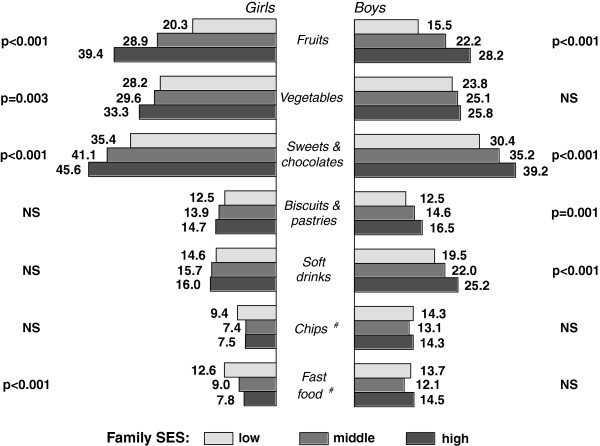
**Crude rates (%) of daily eating of fruits and vegetables, and regular intake of sweets and chocolates, biscuits and pastries, soft drinks, chips and fast food among boys and girls by family SES (pooled data from surveys in 2002, 2006 and 2010)**. p - significance from χ^2 ^test (NS - not significant); ^# ^no data from survey in 2002.

**Figure 3:**
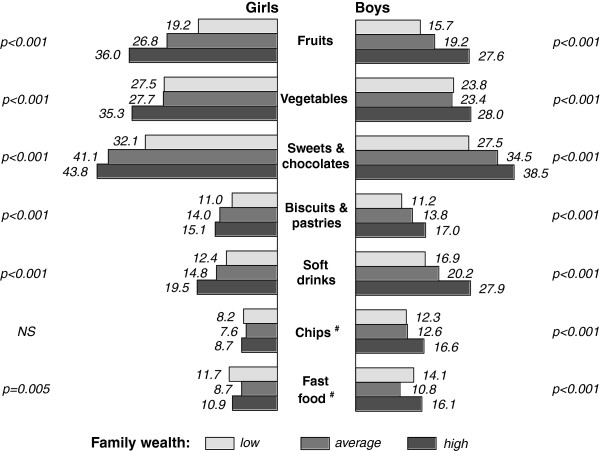
**Crude rates (%) of daily eating of fruits and vegetables, and regular intake of sweets and chocolates, biscuits and pastries, soft drinks, chips and fast food among boys and girls by subjective rating of family wealth (pooled data from surveys in 2002, 2006 and 2010)**. p - significance from χ^2 ^test (NS - not significant); ^# ^no data from survey in 2002.

Analyses of associations between students eating behaviour, their family SES and wealth indicated that there were substantial social inequalities. The prevalence of reported daily fruit and vegetable consumption (among girls only), regular intake of sweets and chocolates, biscuits and pastries (among boys only) and soft drinks (among boys only) increased significantly with respondents' family SES position (Figure 2). Similar differences in food intake prevalence were observed in regards to family wealth groups (Figure 3).

Daily fruit consumption was characterized as the most sensitive variable to family SES and family wealth inequalities. The odds of daily fruit intake for boys and girls living in high SES families were over 2 times greater than for those in lower SES families (OR = 2.15 among boys, and OR = 2.55 among girls). Students living in the most well off families were more likely (boys 1.97 times, girls 2.34 times) to report daily fruit intake than students living in less well off families. Relationships between regular consumption of other foods, family SES and wealth position expressed in ORs are presented in Table 2.

### Food consumption trends

**Figure 4:**
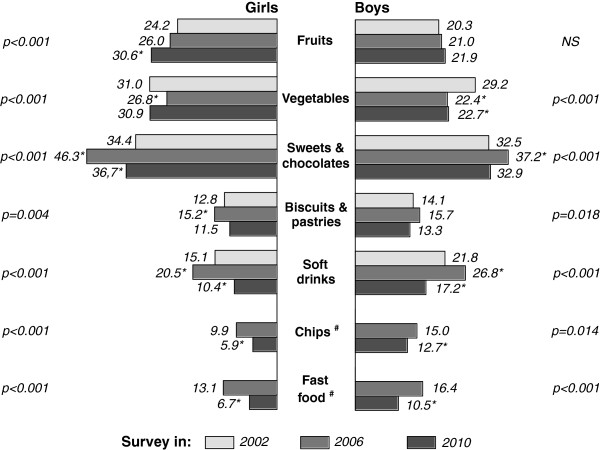
**Crude rates (%) of daily eating of fruits and vegetables, and regular intake of sweets and chocolates, biscuits and pastries, soft drinks, chips and fast food among boys and girls, by survey year (2002, 2006 and 2010)**. p - significance from χ^2 ^test (NS - not significant); * p < .05 in comparison with 2002 (Z test); ^# ^no data from survey in 2002.

Crude rates for daily consumption of fruits, vegetables and other foods were calculated for each survey year (Figure 4). Differences in food consumption rates between study years were significant for all kinds of food; aside from daily fruit intake among boys, several specific trends were revealed too. Comparing 2010 to 2002, the proportion of girls who consumed fruit daily increased from 24.2% to 31.0% (p < 0.001), but the proportion of boys who consumed vegetable daily decreased from 29.3% to 23.1% (p < 0.001). For both sexes, in 2006, we noted an increase in the regular intake of sweets and chocolates, biscuits and pastries, and soft drinks. However, in the next survey (2010) these figures decreased. Between 2006 and 2010 a substantial decrease in regular consumption of chips and fast food was detected as well.

Differences between years of the survey were also shown in the ORs estimated by three logistic models, where 2002 (or 2006 for chips and fast food) were chosen as reference points (Table 3). Results from data analysis with the first model, which adjusted eating behaviour for age only, corresponded to those described above. The second model used data adjusted for age, family SES and subjective rating of family wealth; the third model used data additional adjustments for place of residence and family structure. Comparing the models, it is apparent that adjusting for social variables decreased the OR values for several food consumption trends. A substantial change in likelihoods was achieved by adjusting data for age, family SES and subjective rating of family wealth (model 2), while the impact of additional variables used for adjustment (place of residence and family structure, model 3) caused only minor changes to ORs. Among boys, the change of all social variables used in the present analysis across the 8-year span (from 2002 to 2010) affected the likelihood of daily consumption of fruit by 22.5% (the corresponding OR decreased from 1.11 to 0.86) and the likelihood of daily consumption of vegetables by 8.3% (the corresponding OR decreased from 0.72 to 0.66). Among girls the OR value for daily consumption of fruit in 2010 compared to 2002, shown by model 1, decreased from 1.41 to 1.12 (decreased by 34.0%) and lost its statistical significance, while the impact of social variables change over study period on the likelihood of daily consumption of vegetables was much smaller. Adjusting data for social variables showed a very slight decrease in the likelihoods of regular intake of sweets and chocolates, biscuits and pastries, and soft drinks. There was no data for eating of chips and fast food in 2002; in this respect, ORs trends for these foods were estimated in comparison with the data collected in 2006. Therefore, decreased regular usage of these products between 2006 and 2010 was unlikely to be related to the social determinants.

**Table 3:** Trends of eating habits among school-aged children in Lithuania, 2002-2010: odds ratio (OR) with 95% confidence interval (CI) and p-value for trends

	Daily fruit intake	Daily vegetable intake	Regular intake of sweets and chocolates	Regular intake of biscuits and pastries	Regular drinking of soft drinks	Regular intake of chips^#^	Regular intake of fast food^#^
	OR 95% CI	OR 95% CI	OR 95% CI	OR 95% CI	OR 95% CI	OR 95% CI	OR 95% CI
**Boys:**							
Model 1 * Year of the survey:	p = 0.286	p < 0.001	p < 0.001	p = 0.019	p < 0.001	p = 0.015	p < 0.001
2002	1	1	1	1	1		
2006	1.05 0.92-1.19	**0.70** 0.62-0.79	**1.23** 1.10-1.37	1.13 0.98-1.31	**1.31** 1.16-1.48	1	1
2010	1.11 0.98-1.26	**0.72** 0.64-0.81	1.03 0.92-1.15	0.92 0.79-1.06	**0.74** 0.65-0.85	**0.83** 0.72-0.96	**0.60** 0.51-0.70

Model 2 ** Year of the survey:	p = 0.208	p < 0.001	p = 0.001	p = 0.006	p < 0.001	p = 0.009	p < 0.001
2002	1	1	1	1	1		
2006	0.91 0.80-1.04	**0.66** 0.58-0.74	**1.15** 1.02-1.28	1.06 0.91-1.23	**1.23** 1.08-1.39	1	1
2010	0.89 0.78-1.02	**0.67** 0.59-0.76	0.92 0.82-1.04	**0.83** 0.71-0.98	**0.65** 0.57-0.75	**0.85** 0.73-0.99	**0.58** 0.49-0.68

Model 3 *** Year of the survey:	p = 0.086	p < 0.001	p < 0.001	p = 0.010	p < 0.001	p = 0.009	p < 0.001
2002	1	1	1	1	1		
2006	0.89 0.78-1.03	**0.65** 0.57-0.74	**1.16** 1.03-1.30	1.05 0.90-1.23	**1.22** 1.07-1.38	1	1
2010	**0.86** 0.75-0.99	**0.66** 0.58-0.75	0.90 0.80-1.01	**0.83** 0.71-0.98	**0.63** 0.54-0.72	**0.81** 0.69-0.95	**0.60** 0.47-0.66

**Girls:**							
Model 1 * Year of the survey:	p < 0.001	p < 0.001	p < 0.001	p = 0.004	p < 0.001	p < 0.001	p < 0.001
2002	1	1	1	1	1		
2006	1.10 0.98-1.25	**0.81** 0.72-0.92	**1.65** 1.48-1.86	**1.22** 1.05-1.43	**1.45** 1.26-1.67	1	1
2010	**1.41** 1.25-1.59	1.00 0.89-1.12	**1.15** 1.03-1.29	0.96 0.82-1.13	**0.67** 0.57-0.79	**0.60** 0.49-0.73	**0.49** 0.41-0.59

Model 2 ** Year of the survey:	p = 0.043	p < 0.001	p < 0.001	p = 0.003	p < 0.001	p < 0.001	p < 0.001
2002	1	1	1	1	1		
2006	0.95 0.84-1.08	**0.77** 0.69-0.87	**1.52** 1.36-1.70	1.17 1.00-1.37	**1.35** 1.17-1.56	1	1
2010	1.11 0.97-1.28	0.93 0.83-1.05	1.01 0.90-1.14	0.89 0.76-1.06	**0.62** 0.52-0.73	**0.61** 0.50-0.75	**0.50** 0.41-0.60

Model 3 *** Year of the survey:	p = 0.015	p < 0.001	p < 0.001	p = 0.006	p < 0.001	p < 0.001	p < 0.001
2002	1	1	1	1	1		
2006	0.93 0.82-1.06	**0.78** 0.69-0.88	**1.54** 1.38-1.73	1.15 0.98-1.35	**1.34** 1.16-1.55	1	1
2010	1.12 0.98-1.27	0.96 0.85-1.08	1.02 0.90-1.15	0.89 0.75-1.05	**0.60** 0.50-0.71	**0.62** 0.50-0.75	**0.49** 0.41-0.60

* adjusted for age; ** additionally adjusted for family SES and subjective rating of family wealth; *** additionally adjusted for place of residence and family structure; ^# ^no data from survey in 2002.

## Discussion

The HBSC study is the primary source of data to measure trends in children's health behaviours, including changes in food consumption over time in Lithuania. The data are collected from countrywide representative samples, with questionnaires repeated at regular four-year intervals. Using consistent methodology for subsequent studies, measures are taken to ensure the population is evaluated, and the methods used for dietary surveys, social and economic measures, data processing and analysis are all consistent with prior surveys. The methodology used in this study corresponds with international standards and international comparison, including analysis of dietary intake trend data from varying countries [13,14].

The HBSC study report [9] assembles a comprehensive cross-national picture of the eating habits of young people from 41 countries and regions. According to their nutrition habits, Lithuanian schoolchildren take various positions on the rating scale, when compared with their peers from other countries and regions. Lithuanian children eat fruits and vegetables relatively rarely. Data from 2002, 2006 and 2010 surveys indicate that only 21.1% boys and 27.1% girls had daily intake of fruits; on average, 24.9% of boys and 29.6% of girls reported daily intake of vegetables. For all surveys, national data were significantly lower than the average rates in HBSC countries (in 2005-2006, only 15-year-olds from Greenland reported less consumption of fruits than Lithuanians [9]). Our data on fruit and vegetable consumption were similar to the data from other studies carried out in Lithuania [17,18]. Thus, with regards to fruit and vegetable consumption we conclude that Lithuanian school-aged children consume these products less often. Surely, there could be seasonal explanations as these studies were completed in springtime, when accessibility of fruits and vegetables is reduced in our country, but this limitation was common for all mentioned studies.

In regard to consumption of less healthy food products (sweets and chocolates, biscuits and pastries, soft drinks, chips and fast food), in this cross-national perspective Lithuanian school-aged children should becharacterized positively. The data from the HBSC survey showed very large variations in soft drink consumption between countries. Lithuanian children had one of the lowest reported levels of soft drink consumption [9]. Compared to the EU average, Lithuanian children rarely consumed sweets and soft drinks (Coca-Cola, Sprite or other) on a daily basis in 2001-2002 [19].

It was found that girls of all ages eat fruits, vegetables, sweets or chocolates more often than boys; however, an inverse gender difference exists for consumption of soft drinks, chips and fast food. Daily fruit and vegetable consumption drops significantly between the ages of 11 and 15 years among Lithuanian children. These gender and age specific differences are the case among school-aged children in the majority of HBSC countries [3,20,21] and have also been observed in other studies [11,22,23]. These studies demonstrated that older school-aged children tend to consume less fruits and vegetables than younger school children due to growing peer influence and reduced impact of parents [11,23].

Given the current need to explain the reasons for the healthy and unhealthy food consumption, social determinants should be explored as the main factors that might influence nutrition among school-aged children. The effect of these determinants can be different for different food items [21].

In our study only four social position variables (place of residence, family structure, SES and subjective rating wealth), which were measured by the same methods in all surveys, were included in the analysis. Subjective rating of family wealth, which is a summarizing measure, was used for more detailed analysis and comparison of different social groups of adolescents.

Upon comparing respondents by place of residence, greater daily consumption of fruits was found among both boys and girls living in rural areas, while a more prevalent consumption of vegetables was found among only rural boys. Regular consumption of sweets and chocolates was about 1.5 times more likely among children from rural areas, whereas their peers from urban areas were 1.5 times more likely to eat biscuits and pastries, and to drink soft drinks. On the one hand, such associations might be explained by the difference in the availability of these foods between urban and rural children. Studies in USA, for example, revealed living closer to convenience stores (supermarkets) was associated with increased consumption of crisps, chocolate and white bread [24]. Urban corner shops can be a major risk for children's nutrition because the most frequently purchased items low-nutritive foods and beverages that are energy dense, such as chips, candies and sugar-sweetened beverages [25]. On the other hand, considering the data from Statistics of Lithuania the average disposable income per household per month in urban areas is much higher than in rural areas, although converse consumption expenditure for food is fixed in our country [26]. Consequently, economic differences by place of residence are sharp in Lithuania and can provide one explanation for nutrition inequalities among school-aged children.

Fruit and vegetable consumption increased with family social-economic status and family material wealth (children living in high socioeconomic/rich families were 2 times more likely to eat fruit and vegetables daily than children living in low socioeconomic/poor families). In addition to fruits and vegetables, significant differences between SES groups in sweet and chocolate consumption were also found in our survey. This finding could probably be explained by higher prices of these food products in Lithuania. There is considerable evidence for correlations between low household food budgets and the consumption of cheaper, low-nutritive foodstuffs [11]. Studies confirm that school-children reporting food poverty are less likely to eat fruits, vegetables and brown breads, (odds ratios (ORs) varied from 0.66 to 0.81), and are more likely to eat crisps, fried potatoes and hamburgers (OR varied from 1.20 to 1.62) [27]. Lower fruit and vegetable prices, higher fast food prices, and greater supermarket availability are related to higher fruit and vegetable consumption, especially among teenagers who are at risk for overweight and who are low-to middle-socioeconomic status [28,29].

According to our data, family structure was a significant predictor of nutrition inequalities among Lithuanian adolescents; however, only in cases of reduced chip intake and increased vegetable consumption in girls, who live in intact, as opposed to non-intact, families. Our analysis demonstrated that non-intact families have decreased overall nutrition, due to lower income and home comfort [30,31]. Previous studies reinforce our results, suggesting significant differences exist between "healthy" and "unhealthy" food consumption among children living in intact and non-intact families, which are mainly related to divorce [5,32]. Psychological factors after divorce also have a large influence on a child's nutrition and health [33].

In Lithuania, social factors included in the present analysis, changed significantly during the study period. It is possible these social factors affected the eating habits and health of young people. We recorded more children living in urban areas as well as those living in non-intact families, although positive changes in SES and subjective rating of family wealth were identified. Meanwhile, many macro-social rates changed in our country and most of them turned negatively [9]. The rates of unemployment increased [34] as well as the risk of poverty for families with children [35]; food prices (especially in fruits and vegetables, dairy products and grain) raised in Lithuania over past years [36]. Changes in social environment may have had an impact on the dietary intake of Lithuanians, particularly among children. Monitoring time trends of dietary habits and social determinants is very important for testing such a hypothesis. In a large measure, our study offers such opportunity, as three regular cross-sectional surveys on a representative sample of population were carried out by the same methods of data collection between 2002 and 2010.

Most studies on social inequalities in habitual dietary patterns are based on cross-sectional data collection design [21,37], or under presumption that the differences between SES classes were rather stable over time [38,39]. In this regard, identification of associations in time trends analysis is a difficult task in social epidemiology. However, in the present study, we hypothesized that associations between eating behaviour of children and the survey year were explainable by a differential distribution of social variables across survey years. Adjustment of data across years of the survey, by including social variables into the model, provided empirical evidence for the impact of social inequality changes. Similar approaches have been used in other studies, irrespective of the scientific problem addressed [40,41].

The method of data adjustment for social determinants revealed that the trends of fruit and vegetable consumption, among Lithuanian schoolchildren, were associated with changes in social inequalities over the past decade. The highest decrease in time trends of odds was observed for daily eating of fruits (among boys in 22.5% and among girls in 34.0%, comparing 2010 to 2002). Thus, our findings provide consistent evidence for nutritional inequalities in Lithuanian school-aged children, showing a lower consumption of fruits and vegetable due to socio-economic disadvantages [27,42]. Therefore, nutrition changes need to be assessed in consideration with future social and economic changes.

Evidence suggests that school fruit and vegetable programmes can increase fruit and vegetable consumption among children by as much as 70 percent [4]. These schemes have the added benefit of improving dietary intake of children from low socio-economical backgrounds and reducing health and social inequalities [4,43]. Decline in the consumption of unhealthy foods was observed in 2010 for both sexes. These trends could be explained by the implementation of several health policies and interventions, such as fruit and milk promotional programmes in secondary schools, in Lithuania [44]. In regard to these findings, fruit and vegetable programmes should be high on the political agendas in Lithuania and other countries. Healthy nutrition strategy should be put in practice, since positive changes in child nutrition are expected only when health inequalities in Lithuania are minimized.

### Limitations

The study provides national information about eating behavior and trends among school-aged children in changing social environment. The HBSC questionnaire, which was approved through a series of validation studies, was used for data collection. Despite concerted efforts to obtain reliable data sets for all repeated cross-sectional surveys, data gathered by self-reported food frequency questioning and social assessment remain a matter of great concern. A possible information bias may have been introduced by small changes in item formulations and response categories between surveys [3]. Also, changes in the wording, number and order of response categories influence the participants' responses [45]. In order to avoid any bias related to these changes, data on food consumption from only three surveys in 2002, 2006 and 2010 were used in the present study. For similar reasons, only four social variables were selected to describe the individual social environment of respondents.

The questions on nutrition were designed to provide information on the frequency of food items consumption, but not the quantities consumed. The literature shows varying results regarding the validity of self-reported dietary assessment methods among adolescents [46]. However, a validation study of the food frequency scale, which was used in the present study to assess respondents' eating behavior, showed high test-retest reliability and acceptable validity compared to 24 hour food behaviour checklists and a 7-day food diaries [47]. While it is considered that data generated by food frequency scale may be less suitable for estimating prevalence levels, its applicable for trend analyses is prevalent [3].

One of the major difficulties in monitoring health inequalities among children is the choice of relevant measures for socio-economic status of respondents [48]. Unfortunately, measuring the social environment of respondents in our study was limited to four items, due to previously mentioned reasons. These items can act as determinants in the relationship between household wealth and child's food consumption, but relationships and interactions between these items were not considered. Multivariate analysis would be needed to gain more insight into these relationships, and thus into the underlying mechanisms linking wealth and food consumption among children. Further explorations of possible causes (for example prices, marketing polices, etc) of the observed changes in food intake require a broader approach than is possible in the context of this paper.

Finally, when examining social inequalities, a problem of artifact changes in rates arises if the sizes of the social groups differ between surveys. To reduce such difficulties in measuring health inequalities some authors [48] recommend applying the Relative Index of Inequality, which is an overall measure of the magnitude of inequality across all wealth groups. In the present study, we attempt to solve this problem in part by adjusting data in logistic regression models.

## Conclusions

In summary, our data indicate that Lithuanian school-aged children are not consuming enough fruits and vegetables, meanwhile they might be characterized by moderate consumption of less healthy foods. This phenomenon was quite stable over a period of 8 years. Important social and demographic differences were found in the consumption of both "healthy" and "less healthy" food items among children. In Lithuania, existing socio-economical disadvantages are very important for the nutrition of school-aged children and should be a priority, in order to combat health and social inequalities of children.

Also, the results of this report indicate a need for continued monitoring of eating behaviors and social determinants affecting food consumption among Lithuanian school-aged children. The Lithuanian National Health Council should evaluate the implications for nutrition policy of the results discussed here.

## Competing interests

The authors declare that they have no competing interests.

## Authors' contributions

AZ made substantive intellectual contribution to the conception and design of the manuscript, and carried out statistical analysis. RL and JL were involved in data collection and in drafting of the manuscript. RB made substantial improvements to the manuscript and corrected text. All authors read and approved the final manuscript.

## Pre-publication history

The pre-publication history for this paper can be accessed here:

http://www.biomedcentral.com/1471-2458/12/52/prepub
